# Alterations in Pattern Baldness According to Sex: Hair Metabolomics Approach

**DOI:** 10.3390/metabo11030178

**Published:** 2021-03-18

**Authors:** Yu Ra Lee, Bark Lynn Lew, Woo Young Sim, Jongki Hong, Bong Chul Chung

**Affiliations:** 1 Molecular Recognition Research Center, Korea Institute of Science and Technology, Seoul 02792, Korea; T16627@kist.re.kr; 2 KHU-KIST Department of Converging Science and Technology, Kyung Hee University, Seoul 02447, Korea; 3 Department of Dermatology, Kyung Hee University Hospital at Gangdong, Kyung Hee University, Seoul 05278, Korea; bellotte@hanmail.net (B.L.L.); sim@khnmc.or.kr (W.Y.S.); 4 College of Pharmacy, Kyung Hee University, Seoul 02447, Korea

**Keywords:** hair metabolomics, ultra-performance liquid chromatography-mass spectrometry, pattern baldness, female, male

## Abstract

Pattern baldness has been associated with the male hormone, dihydrotestosterone. In this study, we tried to determine how the overall metabolic pathways of pattern baldness differ in patients and in normal controls. Our study aimed to identify alterations in hair metabolomic profiles in order to identify possible markers of pattern baldness according to sex. Untargeted metabolomics profiling in pattern baldness patients and control subjects was conducted using ultra-performance liquid chromatography-mass spectrometry. To identify significantly altered metabolic pathways, partial least squares discriminant analysis was performed. Our analysis indicated differences in steroid biosynthesis pathway in both males and females. However, there was a remarkable difference in the androgen metabolic pathway in males, and the estrogen metabolic and arachidonic acid pathways in females. For the first time, we were able to confirm the metabolic pathway in pattern baldness patients using hair samples. Our finding improves understanding of pattern baldness and highlights the need to link pattern baldness and sex-related differences.

## 1. Introduction

Pattern baldness is a well-known condition that tends to increase in male hormone secretion [1]. It is suggested that increased formation and exposure of dihydrotestosterone to hair follicles is a possible causative factor of male pattern baldness [2]. The hair growth cycle progresses gradually through the anagen, catagen, and telogen phases [3,4]. Baldness occurs as this cycle rate gradually decreases, and the hair follicles gradually become smaller [5]. Pattern baldness involves a clear progression from the hairline and scalp vertex parts [6,7,8], with decreasing central scalp hair density and gradual thinning of hair. Pattern baldness is of great interest as it can have psychosocial effects in early and severe cases [9]. Female pattern baldness has a postpubescent onset with variable progression in rapidity [10]. Clinical presentation of female pattern baldness differs from that of male pattern baldness [11]. The pathogenesis of female pattern baldness is not as easily recognizable as that in males and its diagnosis is difficult [12]. The causes of female pattern baldness are not as clear as male pattern baldness. Female pattern baldness is probably a genetically determined multifactorial trait and both androgen-dependent and -independent mechanisms may contribute to the phenotype [13]. In addition, the serum testosterone level in female pattern baldness patients is typically normal [14,15,16]. It is significant to note that hyperandrogenemia does not necessarily lead to female pattern baldness [17].

Metabolomics is the scientific approach of comprehensively analyzing metabolites and metabolic pathways in a biological system [18,19] and metabolomics data can be used to detect thousands of features. However, extracting meaningful results and interpretation of metabolomics data is challenging. To overcome dimensionality problems, multivariate statistical analyses are required to arrive at biologically relevant conclusions from a given metabolomics dataset. Untargeted metabolic approaches have focused on collecting data from as many species as possible and reviewing known or unknown metabolic changes [20]. Then, these approaches are used to confirm altered pathways and search for possible biomarkers. Therefore, in this study, we examined whether there is a correlation between metabolic pathways and pattern baldness patients and healthy controls according to sex using untargeted metabolomics.

We conducted experiments using hair samples that are non-invasive and easy to collect. These samples can be stored for long periods of time before analysis without deterioration [21]. Hair analysis can be used to assess current exposure and past episodes related to health and nutrition, even if the action has already been stopped [22]. In particular, hair analysis can be used to construct basic biological matrices for drug testing in forensic toxicology [23]. Till now, research on endogenous compounds in hair has mainly focused on targeting methods for specific compounds such as testosterone, dihydrotestosterone [24,25], cholesterol [26], or cortisol [27].

In this study, hair metabolomics profiling was conducted in pattern baldness patients and normal controls in males and females because of the difference in mechanism of hair loss between the sexes. We aimed to detect altered biochemical pathways that clearly distinguished patients and controls and to investigate the possibility of performing metabolomics using hair samples.

## 2. Results

### 2.1. Metabolic Patterns Detected in Hair Samples of Pattern Baldness Patients

**Figure 1:**
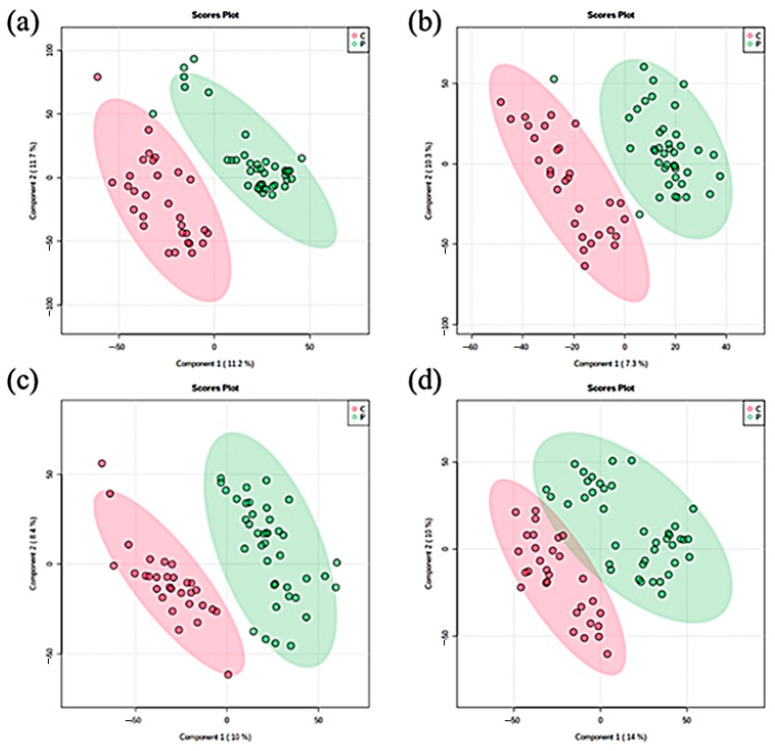
Partial least squares discriminant analysis (PLS-DA) score plots showing discriminant different patterns in controls (red circles) and pattern baldness patients (green circles). (**a**) PLS-DA in positive mode with male groups, (**b**) PLS-DA in negative mode with male groups, (**c**) PLS-DA in positive mode with female groups, and (**d**) PLS-DA in negative mode with female groups.

In male groups, the score plots of the partial least squares discriminant analysis (PLS-DA) were used to separate patients and normal controls in positive mode. The value of multiple correlation coefficient (R2) is an index showing how much data variation is explained by the model, and the value of cross-validated R2 (Q2) is an index showing the prediction diagram of the model when a new variable is applied. We calculated R2 and Q2 to check the suitability and predictability of the model, by setting the missing data value at run time of the model to 60%. The score plot of PLS-DA with positive mode showed an accuracy of 98%, R2 of 0.93 and Q2 of 0.76 (Figure 1a). In negative mode with male groups, score plots were also clearly separated. The accuracy of PLS-DA was 96.7%, that of R2 was 0.96, and that of Q2 was 0.78 (Figure 1b).

Female groups were also clearly separated in ionization mode. The score plots of PLS-DA in positive mode showed an accuracy of 97.7%, those of R2 showed an accuracy of 0.98, and those of Q2 showed an accuracy of 0.77 (Figure 1c). In negative mode, PLS-DA score plots showed an accuracy of 97%, those of R2 showed an accuracy of 0.96, and those of Q2 showed an accuracy of 0.78 (Figure 1d).

**Figure 2:**
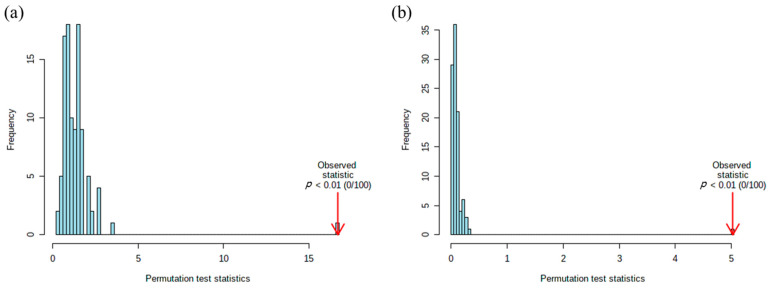
Permutation test to validate partial least squares discriminant analysis score. Permutation numbers were set to 100. X-axis showed the permuted class labels and Y-axis shows optimal number of components by cross validation in (**a**) male groups and (**b**) female groups.

Cross validation method was applied 10-fold CV. We also performed the permutation test to confirm whether the PLS-DA data were statistically significant. We used the ratio of the sum of the squares between and the sum of squares within (B/W-ratio). As shown in Figure 2, the blue bar shows the original samples. The farther right it is to the position of the red arrow, the more significant is the separation between groups [28].

On the basis of variable importance in projection (VIP) values and *t*-test of the metabolites of the PLS-DA model, possible biomarkers that could distinguish patients and normal controls were selected as shown in Table 1 with male groups and in Table 2 with female groups. In male groups, 42 metabolites met these criteria (positive mode: 39 of 1171 metabolites, negative mode: two of 109 metabolites) with VIP > 1 and *p* < 0.05. Whereas in female groups, 26 metabolites met these criteria (positive mode: 20 of 260 metabolites, negative mode: 6 of 109 metabolites). We corrected all the missing metabolite entries and obtained a total of 1018 values (626 values in male groups and 392 values in female groups) with zero. As shown in Table 1 and Table 2, we calculated fold-change (FC) values representing the difference between patients and controls. We calculated the FC values using the average of each metabolite peak area: (average values obtained from pattern baldness patients)/(average values obtained from normal controls). If the FC value was greater than 1, the metabolites were upregulated in patient groups.

**Figure 3:**
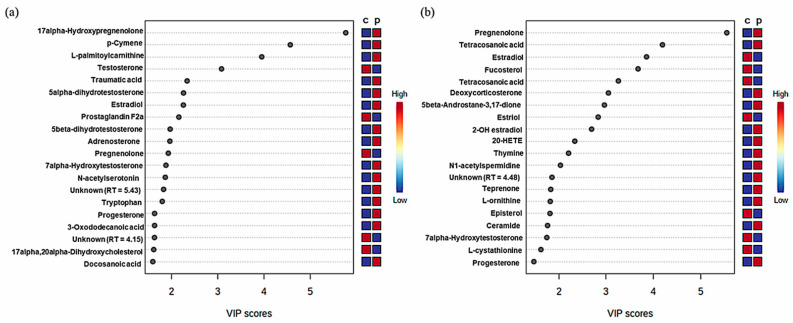
The top 20 scores of variable importance in projection (VIP) values. On the left part of the graph, significant difference of metabolites are indicated. The main graph represents the VIP scores. On the right part of the graph, dark red indicates high abundance and dark blue indicates low abundance, respectively. (**a**) Male groups and (**b**) female groups; c, normal controls; p, pattern baldness patients.

The colored boxes on the right indicate the relative concentrations of the corresponding metabolite in each group. We used MetaboAnalyst version 5.0 software to confirm top 20 scores of variable importance in projection (VIP) values visually (Figure 3). We could visually confirm the alterations of metabolites divided in each row, separated into upregulated or downregulated metabolites.

### 2.2. Metabolic Pathway Analysis

**Figure 4:**
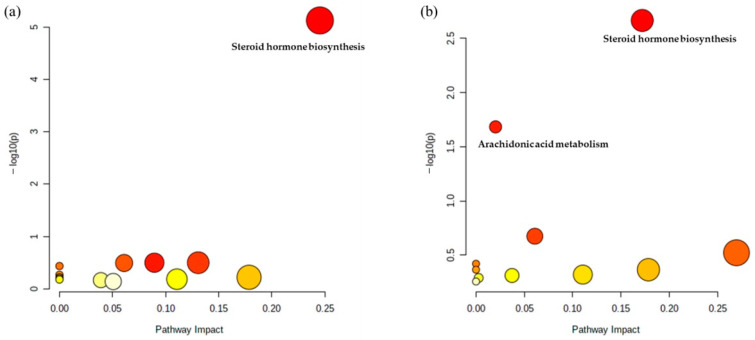
Differentiated metabolic pathways between patient groups and controls can be visually confirmed. Dark red indicates that the pathways were significantly different, with red circles indicating more significance than yellow circles. Light yellow means a less significant than yellow. The Y-axis represents the –log p values, whereas the X-axis represents the pathway impact values. (**a**) Pathway analysis based on 42 metabolites in male groups from an untargeted analysis and (**b**) pathway analysis based on 26 metabolites in female groups from an untargeted analysis.

Among the detected compounds, pathway analysis was conducted with 38 metabolites in male groups and 24 metabolites in female groups using MetaboAnalyst 5.0 library. As shown in Figure 4a, pathway analysis was conducted in male groups to confirm the most differed metabolic pathways between male pattern baldness patients and male controls. As shown in Figure 4b, similarly, pathway analysis was conducted and used to identify the most differed metabolic pathways between female pattern baldness patients and female controls.

## 3. Discussion

In this study, untargeted metabolomics analysis was conducted to identify multiple differentially altered pathways in pattern baldness patients and controls using UPLC-MS. In addition, we divided subjects according to sex to determine the metabolic pathways that differ in pattern baldness. Our novel metabolomics approach employed hair samples from patients with pattern baldness for the first time. The most significantly altered pathway was steroid hormone biosynthesis in both males and females; female groups showed that arachidonic acid metabolism was altered between patients and control groups.

**Figure 5:**
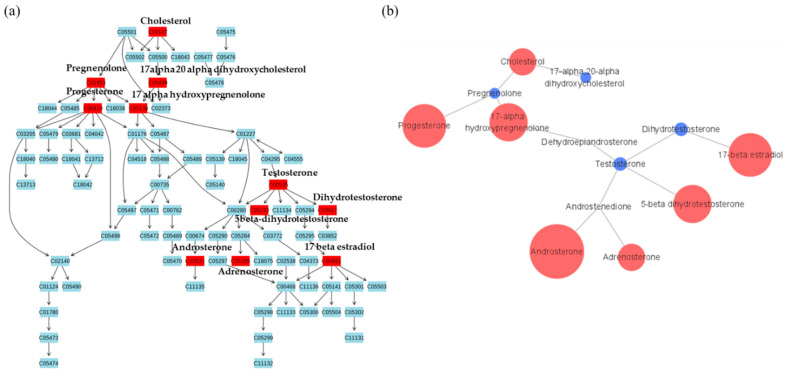
Metabolites with differences between male groups in steroid metabolic pathway. (**a**) Metabolites in red indicate significant differences between groups and (**b**) the pathway mapping of metabolites and relative quantitation between male pattern baldness patients and male controls. The larger the circle, the larger the relative quantitative value. Red circles indicate higher metabolite concentrations in the patient group and blue circles indicate higher metabolite concentrations in the control group.

In male groups, steroid hormone biosynthesis and androgen metabolism were significantly altered (Figure 5a). To show a more accurate correlation between patients and control groups, Figure 5b presents relative quantitation using cytoscape. As androgens are correlated with male pattern baldness, especially testosterone and dihydrotestosterone, their analysis is useful for pattern baldness. We have conducted previous studies on hair loss with respect to genetic factors such as androgens [24,25,29]. This study showed altered levels of downregulated testosterone levels and upregulated dihydrotestosterone levels in hair samples between pattern baldness patients and controls in males. High levels of dihydrotestosterone are known to occur in patients with hair loss [30]. However, progesterone receptor expression does not significantly increase or decrease in in mesenchymal cells in male pattern baldness [31].

In addition to the well-known steroid pathways, we analyzed whether neurotransmitters exhibited significant changes in these groups. Upregulation of *N*-acetylserotonin, dopamine, metanephrine, and tryptophan were observed in different metabolic pathways between male patients and male controls. This may be due to the stress caused by hair loss [32]. Male pattern baldness is known to cause significant psychosocial effects in patients [33]. However, more research is needed because the direct quantitative analysis of hair loss related to psychosocial effects has not yet been studied.

**Figure 6:**
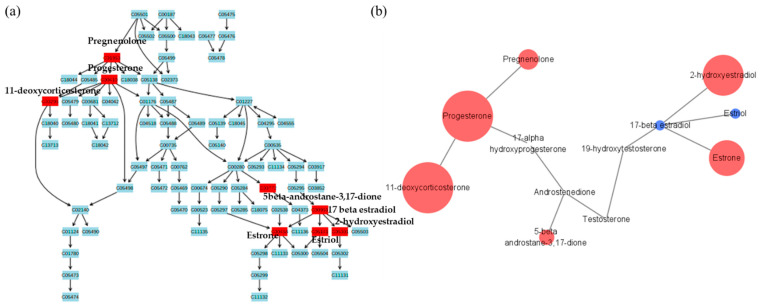
Metabolites with differences between female groups in steroid metabolic pathway. (**a**) Metabolites in red indicate significant differences between groups and (**b**) the pathway mapping of metabolites and relative quantitation between female pattern baldness patients and female controls. The larger the circle, the larger the relative quantitative value. Red circles indicate higher metabolite concentration in the patient group and blue circles indicate higher metabolite concentration in the control group.

In female groups, steroid metabolism was also altered between patients and controls. However, unlike males, it was confirmed that there were significant changes in estrogen hormones and their metabolites in females (Figure 6). Estrogen plays an important role in retaining hair strength by activating hair growth factors and inhibiting hair loss factors [34]. It also reduces the amount of 5α-dihydrotestosterone [35]. Thus, estrogen helps to maintain the hair status in women with hair loss [36]. The ratio of estradiol and free testosterone and the ratio of estradiol to dehydroepiandrosterone sulfate were significantly lower in female pattern baldness patients [37]. Various therapeutics have been employed for the treatment of female pattern hair loss but treatment outcomes are not always satisfactory [38]. There are also studies confirming the efficacy of estradiol as a new treatment for female hair loss [39]. However, there are few studies related to female pattern baldness; therefore, more research is required.

We found that arachidonic acid metabolism differed between patients and control groups in females. Leukotriene B4 and 20-hydroxyeicosatetraenoic acid (20-HETE) were found to be higher in female patients. Arachidonic acid metabolism is correlated with hair growth and plays a role in promotion of hair growth by increasing the expression of growth factors in human dermal papilla cells and enhancing follicle proliferation [40]. 20-HETE has a wide range of effects on the vascular system, including the regulation of blood vessels. However, since leukotriene B4 has not been studied with relation to hair loss, further research is needed.

Prostaglandin F2α was altered in both males and females and was lower in patient groups. Prostaglandin F2α and its products promote hair growth in the anagen stage and are involved in initiating hair regrowth [41]. Latanoprost (analogue of prostaglandin F2α), which is used for the treatment of glaucoma and ocular hypertension, stimulates hair growth in pattern baldness with topical daily application for five months [42]. We confirmed it to be downregulated in both male and female patients. This suggests that there is an association between a decrease in prostaglandin F2α levels and hair loss, which is not sex-related. However, accurate quantitative analysis is required for detailed pathogenesis of pattern baldness.

In addition, we found that ornithine was significantly altered in both male and female groups and was upregulated in both patient groups. Ornithine is known to be involved in the biosynthesis of polyamines and cell proliferation. Ornithine decarboxylase inhibitor prevents hair loss and partially normalizes skin histology [43]. In particular, ornithine decarboxylase, a key enzyme in the synthesis of polyamines, is expressed in the vicinity of the hair follicle bulge [44]. Therefore, we conducted analysis of polyamines, which are biosynthesized via ornithine, and their relation to different types of hair loss such as androgenic alopecia and alopecia areata [45]. We also conducted polyamine profiling of different scalp regions [46].

We confirmed that well-known metabolic pathways such as steroid biosynthesis were altered between pattern baldness patients and normal controls. However, the differences in androgens were remarkable in males, and differences in estrogen hormones were clear in females. In addition, in female groups, arachidonic acid metabolism pathways, which are related to inflammation, were altered. We also confirmed that the changes in ornithine levels were common to males and females. However, a comparative study of subjects of different races and a larger cohort is needed for a complete understanding of the pathways involved in baldness pattern. Though validating biomarkers were not used in this study, the results of our study can be used to identify possible markers. In the future, we plan to conduct quantitative analysis on various metabolites that differed significantly in this study.

In conclusion, we offer a study of the extensive metabolic alterations associated with pattern baldness according to sex. Further, our study suggests that obtaining hair samples from subjects is non-invasive and easy and can be used to perform metabolomics study of pattern baldness. We demonstrated that pattern baldness was related to steroid hormone biosynthesis in both sexes. However, androgen metabolism was found to be significantly altered in males, whereas the estrogen metabolism was found to be significantly altered in females. In addition, it was confirmed that metabolic pathways of arachidonic acid were significantly altered in females. Thus, we confirmed that various metabolic pathways are involved in the cause of pattern baldness and the pathogenesis of hair loss differs according to sex, suggesting that further studies on the correlation between pattern baldness and various metabolites according to sex are needed.

## 4. Materials and Methods

### 4.1. Patients

Untargeted profiling was conducted using 140 human hair samples, i.e., male pattern baldness patients (*n* = 40, aged 10 to 19 years, mean 15.9) and female pattern baldness patients (*n* = 40, aged 15 to 44 years, mean 27.7), male controls (*n* = 30, aged 20 to 26 years, mean 22.1), and female controls (*n* = 30, aged 21 to 43 years, mean 30.1). Exclusion criteria included the presence of hormone-related disease other than pattern baldness or severe inflammatory disease. The inclusion criteria enabled the selection of pattern baldness who did not meet the exclusion criteria. Controls did not have any known disease and did not take any medication.

### 4.2. Hair Sample Collection

Hair samples were collected from scalp hair as close to the scalp as possible with scissors. These hair samples were obtained from subjects at Kyung Hee University at Gangdong. Thin and short miniaturized hair of pattern baldness patients were acquired. The collected hair included normal terminal hair that were thinner than the normal. Hair samples were stored at room temperature until analysis. The study was approved by the Ethics Committee of the Kyung Hee University Hospital at Gangdong (IRB No. 2007-03-004). Written informed consent was acquired from all participants prior to sample collection, and consent was acquired from a parent or legal guardian prior to participation by minors in this study.

### 4.3. Chemicals and Materials

All chemicals we used were obtained from Sigma Aldrich (St. Louis, MO, USA). High performance liquid chromatography grade isopropyl alcohol, methanol, and acetonitrile were obtained from Burdick & Jackson (Muskegon, MI, USA). Water was purified using Millipore Milli-Q Water Purification System (Bedford, MA, USA). Homogenization of hair samples was performed using TissueLyser from Qiagen (West Sussex, UK).

### 4.4. Sample Preparation

Hair samples were cleaned with water and isopropyl alcohol and dried at 60 °C. After drying, the samples were pulverized and extracted using methanol at 40 °C. Before analysis, the samples were centrifuged using Ultrafree-MC-VV centrifugal filters (Burlington, MA, USA) at 1100× *g* for 5 min.

In order to validate system stability and repeatability, pooled quality control (QC) samples were prepared by mixing equal amounts of samples. To ensure system stability, 10 QC samples were injected prior to analysis, and solvent blank and QC samples were injected after every 10 sample injections to verify system reproducibility. 

### 4.5. Experimental Conditions

The experimental conditions were the same as those previously established [47,48]. Table 3 shows detailed information of experimental conditions used. The data from these instruments were collected using MassLynx 4.1 software from Waters (Milford, MA, USA).

### 4.6. Statistical Analysis

Statistical analysis was performed as described previously [47,48]. Retention time and *m/z* were calculated using MassLynx 4.1 software. Raw data were collected and processed with baseline correction, scaling, peak alignment, and matrix manipulation using MassLynx. Chromatographic peaks, ion intensity identification, and data matrix constriction were also analyzed using MassLynx. Subsequently, MassTRIX was used to search *m/z* values of possible markers with a maximum error of 0.05 Da. The data of divided IDs and compounds were attached to other data spreadsheets. Accurate mass queries were raised in compound databases such as Metlin, Human Metabolome Database, PubChem, ChemSpider. Fragmentation patterns were found in spectral databases such as MassBank and NIST2014 for structural identification with molecular formulas. MetaboAnalyst 5.0 software (McGill University, Montréal, QC, Canada) was used to perform PLS-DA analysis. Candidate markers were obtained with the conditions of VIP > 1, pathway search results, and *p* < 0.05. Student’s *t*-test was performed using SPSS Statistics 18 software (IBM, Armonk, NY, USA). *p*-values were corrected using the Benjamini and Hochberg false discovery rate adjustment.

## Figures and Tables

**Table 1:** Differentially regulated metabolites in patients with pattern baldness and normal controls in male groups.

Metabolites	Related Pathways	VIP	*p*-Value	FC	Regulation
l-Ornithine	Arginine and proline metabolism	1.1985	0.003	1.5919753	Up
Cholesterol	Steroid hormone biosynthesis	1.0319	0.004	2.0443951	Up
Progesterone	Steroid hormone biosynthesis	1.8311	<0.0001	10.00754	Up
Androsterone	Steroid hormone biosynthesis	1.0189	<0.0001	23.17902	Up
Prostaglandin F2alpha	Arachidonic acid metabolism	3.0556	0.001	0.2634053	Down
Tryptophan	Unknown	2.2307	<0.0001	2.4243554	Up
Estradiol	Steroid hormone biosynthesis	3.8042	<0.0001	9.6410812	Up
*N*-Acetylserotonin	Tyrptophan metabolism	2.237	<0.0001	15.45131	Up
5-Aminopentanamide	Unknown	1.2417	<0.0001	48.086484	Up
Unknown (RT = 5.21)	Unknown	1.0823	0.023	3.6537206	Up
Lanosterol	Steroid biosynthesis	1.1285	0.038	0.488275	Down
Pregnenolone	Steroid hormone biosynthesis	2.3772	0.001	0.4563306	Down
2-Methylmaleate	Unknown	1.2134	<0.0001	2.6569257	Up
l-Cystathionine	Cystein and methionine metabolism	1.2592	<0.0001	0.346639	Down
3-Oxododecanoic acid	Unknown	1.828	<0.0001	8.1830723	Up
Nonane-4,6-dione	Unknown	1.4105	0.015	0.0427089	Down
Dodecanoic acid	Fatty acid biosynthesis	1.6129	0.005	27.655866	Up
l-Palmitoylcarnitine	Fatty acid degradation	4.6032	<0.0001	15.272926	Up
Dopamine	Tyrosine metabolism	1.4983	<0.0001	28.58511	Up
5alpha-Dihydrotestosterone	Steroid hormone biosynthesis	4.0064	0.004	12.736311	Up
Diethyl 2-methyl-3-oxosuccinate	Unknown	1.4048	<0.0001	16.252694	Up
17alpha-Hydroxypregnenolone	Steroid hormone biosynthesis	11.727	<0.0001	5.4864425	Up
Adrenosterone	Steroid hormone biosynthesis	2.4995	<0.0001	2.4143371	Up
17alpha,20alpha-Dihydroxycholesterol	Steroid hormone biosynthesis	1.6553	0.002	0.1035019	Down
l-Metanephrine	Tyrosine metabolism	1.2238	<0.0001	211.3841	Up
Tetradecanoic acid	Fatty acid biosynthesis	1.126	<0.0001	11.466312	Up
*p*-Cymene	Unknown	7.1135	<0.0001	24.273712	Up
Unknown (RT = 3.76)	Unknown	1.264	0.011	2.7952495	Up
Unknown (RT = 4.15)	Unknown	1.7684	<0.0001	0.4185244	Down
Docosanoic acid	Unknown	1.4008	<0.0001	6.895777	Up
Humulene	Unknown	1.2381	<0.0001	24.509825	Up
beta-Citronellol	Unknown	1.2889	<0.0001	2.2233273	Up
Thymol	Unknown	1.2472	0.02	6.2627942	Up
Tridecane	Unknown	1.4906	0.013	5.6917082	Up
4-Propylphenol	Unknown	1.2452	<0.0001	0.3275366	Down
Tetraethylenepentamine	Unknown	1.6745	<0.0001	8.9096476	Up
Traumatic acid	Unknown	4.0178	0.007	3.5804527	Up
Unknown (RT = 5.43)	Unknown	2.2358	<0.0001	339.30032	Up
Neocnidilide	Unknown	1.4039	0.002	1.4515461	Up
Testosterone	Steroid hormone biosynthesis	4.334	0.002	0.4662096	Down
7alpha-Hydroxytestosterone	Unknown	2.3012	<0.0001	1.8760545	Up
5beta-Dihydrotestosterone	Steroid hormone biosynthesis	2.5426	0.01	6.9586924	Up

**Table 2:** Differentially regulated metabolites in patients with pattern baldness and normal controls in female groups.

Metabolites	Related Pathways	VIP	*p* Value	FC	Regulation
l-Ornithine	Arginine biosynthesis	1.3668	<0.0001	11.910219	Up
Thymine	Pyrimidine metabolism	1.9371	0.025	15.622769	Up
Progesterone	Steroid hormone biosynthesis	1.1846	<0.0001	17.966704	Up
N1-Acetylspermidine	Unknown	1.8138	<0.0001	5.4335943	Up
Prostaglandin F2alpha	Arachidonic acid metabolism	1.0325	0.045	0.5604433	Down
Estrone	Steroid hormone biosynthesis	1.7482	<0.0001	10.18276	Up
Octadecanal	Unknown	1.14	0.001	4.7250936	Up
Pregnenolone	Steroid hormone biosynthesis	9.4143	<0.0001	6.1234272	Up
l-Cystathionine	Glycine, serine and threonine metabolism	1.2179	<0.0001	0.3661377	Down
Unknown (RT = 5.22)	Unknown	1.1014	<0.0001	7.8823964	Up
5beta-Androstane-3,17-dione	Steroid hormone biosynthesis	2.7125	<0.0001	3.8285677	Up
7alpha-Hydroxytestosterone	Unknown	1.273	<0.0001	0.3856094	Down
Tetradecanoic acid	Fatty acid biosynthesis	6.6464	<0.0001	6.082907	Up
Unknown (RT = 4.48)	Unknown	1.6103	0.002	1.4591549	Up
Tetracosanoic acid	Unknown	4.0729	0.007	0.6894739	Down
Fucosterol	Unknown	4.3796	0.011	0.7134959	Down
Ribalinium	Unknown	1.108	<0.0001	4.9206557	Up
Teprenone	Unknown	1.5799	<0.0001	9.3409894	Up
Episterol	Steroid biosynthesis	1.3281	0.043	0.4078797	Down
Estriol	Steroid hormone biosynthesis	2.5591	<0.0001	0.5797319	Down
Ceramide	Sphingolipid metabolism	1.2892	0.01	9.651607	Up
Estradiol	Steroid hormone biosynthesis	5.5694	0.04	0.8441624	Down
Leukotriene B4	Arachidonic acid metabolism	1.2626	<0.0001	22.611004	Up
11-Deoxycorticosterone	Steroid hormone biosynthesis	3.5377	<0.0001	18.558709	Up
2-OH-Estradiol	Steroid hormone biosynthesis	2.3525	0.02	13.21151	Up
20-HETE	Arachidonic acid metabolism	2.0165	0.02	1.3674874	Up

**Table 3:** Liquid chromatography-mass spectrometry instrumental conditions.

	**LC Condition**		
Instrument	ACQUITY™ ultra-performance liquid chromatography		
Column	ACQUITY UPLC BEH C18 (2.1 × 100 mm, 1.7 μm)		
Column Temperature	40 °C		
Sampler Temperature	4 °C		
Injection Volume	5 μL		
Flow Rate	0.35 mL/min		
Mobile Phase	A: 0.1% formic acid water		
	B: 0.1% formic acid acetonitrile		
Gradient System	Time (min)	%A	%B
	0	95	5
	3	50	50
	10	5	95
	11.5	5	95
	12	95	5
	14	95	5
	**MS Condition**		
Instrument	Q-Time of flight Premier™ quadrupole/ time-of-flight hybrid mass spectrometer		
Scan Type	Full scan (*m/z* range: 50–1200)		
Cone Voltage	2.5 kV		
Capillary Temperature	120 °C		
Desolvation Temperature	350 °C		
Cone Gas Flow	30 L/h		
Capillary Voltage	2.5 kV		
Desolvation Gas Flow	600 L/h		

## Data Availability

The data presented in this study are openly available. Data available in a publicly-accessible repository.
